# Red and blue laser light drives photosynthesis through dynamic changes of stomatal aperture

**DOI:** 10.3389/fpls.2026.1781728

**Published:** 2026-03-03

**Authors:** Koichi Yoshi, Masaaki Takahashi, Michiya Negishi

**Affiliations:** Research Center for Agricultural Robotics, National Agricultural and Food Research Organization (NARO), Tsukuba, Ibaraki, Japan

**Keywords:** controlled environment agriculture, laser diodes (LDs), photosynthetic induction, plant factories with artificial light (PFALs), stomatal conductance, ultradian oscillations

## Abstract

Amid the growing global demand for sustainable crop production, plant factories with artificial lighting (PFALs) have gained attention as the systems provide stable, optimal growth environments for crops, and are largely unaffected by climate change. However, a limitation of widely used light-emitting diodes (LEDs) in PFALs is their decreased energy conversion efficiency at high output levels, prompting the search for more efficient light sources. This study focused on laser diodes (LDs), which has shown superior energy conversion efficiency, as an alternative. We developed an LD lighting system capable of mixing red and blue light at arbitrary ratios and mounted it onto a commercial gas-exchange measurement system. Using this system, photosynthetic parameters in rice were obtained under conditions of red light alone and as well as combined red and blue light illumination. Under red LD illumination alone, steady-state CO_2_ assimilation rate, stomatal conductance, and transpiration rate in rice were significantly higher than those under red LED illumination, whereas intrinsic water-use efficiency decreased due to a relatively greater increase in stomatal conductance. Notably, stomatal conductance and transpiration rate exhibited pronounced temporal oscillations with a period of approximately 900 s, which closely corresponded to oscillations in stomatal aperture confirmed by microscopic observations. Under combined red and blue LD illumination, steady-state photosynthetic parameters did not differ significantly from those under LED illumination; however, the dominant oscillatory frequency observed under red LD alone was not detected, and some photosynthetic capacity parameters tended to decline. Furthermore, CO_2_ response analyses revealed that, despite lower CO_2_ assimilation, stomatal conductance responded more strongly to changes in intercellular CO_2_ concentration under combined red and blue LD illumination. Taken together, these results demonstrate that LD lighting, particularly red LD, enhances stomatal dynamics and induces characteristic oscillatory behavior compared with LED lighting. While red LD appears to be a promising cultivation light source for PFALs capable of maintaining high photosynthetic activity, the physiological impacts associated with blue LD, including potential reductions in photosynthetic capacity, require further study to optimize blue-light proportions for rice cultivation.

## 1 Introduction

Since the 1950s, the frequency and intensity of extreme temperature events, including heatwaves,as well as droughts and floods, have increased because of human-induced greenhouse gas emissions (Masson-Delmotte et al., 2021; Furtak and Wolińska, 2023). These increasing climate changes highlight the need for agricultural systems that can maintain stable production under variable and unpredictable environmental conditions. Climate-resilient agricultural technologies that enable stable crop production under changing environmental conditions include Controlled‐Environment Agriculture (CEA), which allows crop cultivation under controlled environmental conditions, such as temperature, humidity, CO_2_ concentration, lighting and irrigation (Sagheer et al., 2020; Chen et al., 2025). CEA encompasses plant factories with artificial lighting (PFALs) that enable advanced environmental control and are designed for highly efficient commercial production under artificial light (Kozai, 2013). The PFALs can deliver up to 10 times the yield per unit area of greenhouse cultivation, and over 100 times in open field farming (Benke and Tomkins, 2017; Kozai, 2018). However, the initial cost per unit area in PFALs can be up to 10 times that of greenhouses and 100 times that of open-field cultivation (Kozai, 2018), and operating costs per unit area can reach up to 11 times those of greenhouses (Harbick and Albright, 2016). Within PFALs operating costs, lighting represents the largest share, accounting for approximately 43% to 80% of total running costs (Ohyama et al., 2002; Kozai, 2013; Harbick and Albright, 2016).

LED lighting, which currently dominates lighting systems in PFALs, offer superior luminaire efficiency compared to conventional technologies such as metal halide (MH) lamps, high pressure sodium (HPS) lamps and fluorescent lamps. Under simulated greenhouse conditions providing a photosynthetic photon flux density (PPFD) of 300 µmol m^−2^ s^−1^, when compared with 1000 W HPS and MH lamps, eight of the ten LED lightings tested exhibited higher luminaire efficacy than the HPS lamp, and all ten outperformed the MH lamp (Radetsky, 2018). In lettuce cultivation experiments at PPFDs of 270 and 570 µmol m^−2^ s^−1^, red–blue LED lighting was shown to achieve equivalent yields to fluorescent lamps while using only half the incident energy (Cammarisano et al., 2021). However, although LED exhibits high power‐conversion efficiency (PCE) at low output levels, its PCE is known to decline sharply with increasing current density, called “efficiency droop” (Wierer et al., 2013; Wierer and Tsao, 2015), requiring the development of alternative light sources better suited to PFALs that demands high PPFD per unit area.

Laser diodes (LDs) are light sources with strong directionality, coherence and monochromaticity, capable of delivering high-intensity irradiation over narrow areas (Ferraz et al., 2007). Unlike LEDs, LDs did not suffer from efficiency droop at elevated current densities (Liu et al., 2023), making them promising candidates for cultivation lighting in PFALs. In fact, some reports suggested that LDs have advantages in aspect of plant growth. For early growth promotion, laser irradiation for 20 days has been shown to increase yield by over 10% in rice seedlings compared with natural light alone (Minglai et al., 2024). As supplemental lighting for vegetable cultivation, LDs have been found to enhance photosynthesis compared with white LED lighting in sweet pepper leaves (Hu et al., 2007), further highlighting the potential of LDs for indoor horticultural applications. Both red and blue wavelengths are efficient in photosynthesis, as shown by the action spectrum of CO_2_ assimilation, which displays broad maxima in the red and blue wavelength regions (McCree, 1971). However, most LD‐based cultivation systems rely exclusively on not blue LD illumination but red LD illumination (Yamazaki et al., 2002; Hu et al., 2007; Li et al., 2025), and the effects of LD lighting on the induction kinetics of photosynthesis remain poorly understood.

In this study, we constructed an LD lighting system capable of mixing red and blue LDs at arbitrary ratios and precisely evaluated the effects of red and blue LD illumination on photosynthetic induction in rice.

## 2 Materials and methods

### 2.1 Plant samples and growing methods

In this study, 4-week-old rice plants (Oryza sativa cv. Koshihikari) were used because laser irradiation has been reported to alleviate salt stress in rice (Cheng et al., 2025), implying a potentially high physiological sensitivity to laser light. The seeds were first sterilized completely in 70% ethanol for 30 s, followed by an additional sterilization step with 50% sodium hypochlorite for 30 min. After sterilization, the seeds were rinsed eight times with distilled water to remove residual sterilizing agents. The sterilized seeds were then placed on filter paper moistened with distilled water in 9-cm Petri dishes. The dishes were kept in an incubator (FCI-280, AS ONE Co., Tokyo, Japan) in complete darkness at 30°C for three days to promote germination. On the fourth day, LED illumination was initiated at a light intensity of 100 μmol m^−2^ s^−1^ and maintained for two days. Subsequently, individual seedlings were transplanted into urethane blocks (2.2 cm × 2.2 cm × 2.5 cm) at a density of 20 seedlings per 100 cm². The urethane blocks were soaked in hydroponic nutrient solution (Otsuka-A & B, Inochio Holdings Inc., Aichi, Japan) to prevent desiccation, and the seedlings were cultivated in a growth chamber (NK Systems, Nippon Medical & Chemical Instruments Co., Ltd., Osaka, Japan) until the fourth week after sowing. The growth chamber, equipped with cool-white fluorescent lamps providing broad-spectrum white light, was maintained at 70% relative humidity (RH) with a photosynthetically active photon flux density (PPFD) of 300 µmol m^−2^ s^−1^, a CO_2_ concentration of 400 µmol mol^−1^ and 26°C in a 14 h light period. The nutrient solution (EC 1.2 dS m^−1^) was replaced once a week.

### 2.2 New LD light source

**Figure 1:**
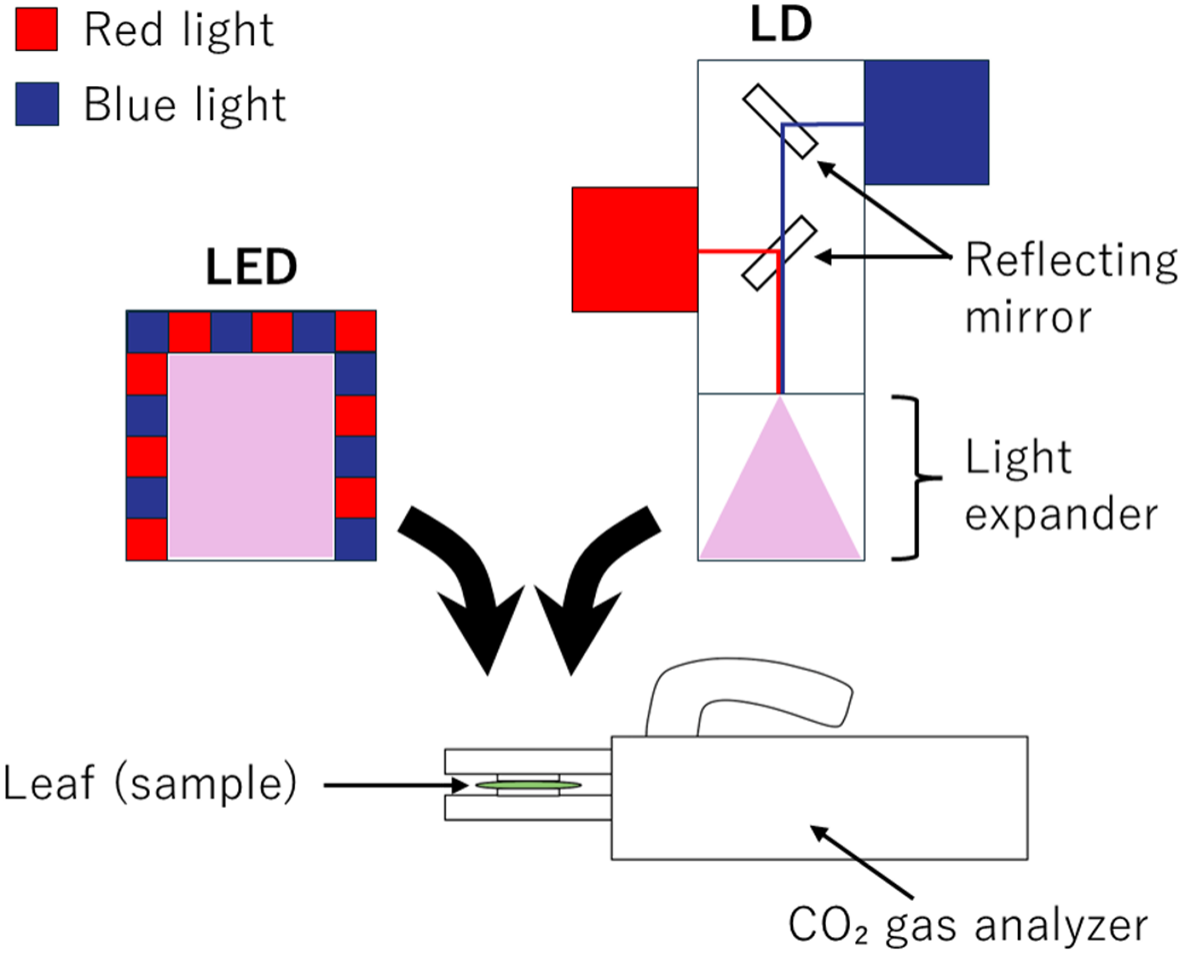
The overview of the equipment used in this study. To investigate whether the type of light source affects leaf-level photosynthetic parameters and plant growth parameters, a LD illumination system was constructed. The CO_2_ gas analyzer is typically equipped with red and blue LED light sources. However, in our experiment, a custom LD light source with blue and red light was designed to be attachable to the CO_2_ gas analyzer.

To investigate the effects of LDs and LEDs on photosynthesis, we introduced a novel LD-based light source (ALT-9H59, ALT Inc., Tokyo, Japan). This unit is designed to be interchangeable with the standard LED light module supplied with the LI-6800P gas-exchange system (LI-COR, Lincoln, NE, USA) and can be mounted onto the chamber head, enabling uniform illumination over a 1 cm × 3 cm leaf area (**Figure 1**). The light source consists of two LD modules: a red LD with a peak wavelength at 660 nm and a blue LD with a peak at 452 nm. These two light sources can be combined at every ratio to achieve desired spectral compositions. While LDs inherently emit highly directional light, the light emitted from this device is diffused by an optical expander to ensure even illumination across the leaf surface; however, the angle between the optical axis and the outermost edge of the irradiated leaf area is limited to approximately 8°.

### 2.3 Wavelength measurement of light sources

The spectral characteristics of each light source were measured using a spectrometer (Ocean SR4, Ocean Optics, CA, USA). Spectral data were collected at 1 nm intervals across the 350–800 nm range while each light source was turned on. Subsequently, background noise was measured with the light source turned off. Spectral intensities were expressed as relative values by normalizing to the peak intensity (set to 1). For each light source, the full width at half maximum (FWHM) was calculated as a measure of peak sharpness, defined as the wavelength interval between the two points on either side of the peak at which the spectral intensity equals half of the maximum.

### 2.4 Gas exchange measurement

Rice plants were dark adapted overnight prior to measurement. Fully expanded third or fourth leaves from 4-week-old seedlings were enclosed in the gas exchange chamber of the measurement system. Only the enclosed leaf area inside the gas-exchange chamber was illuminated during measurements, while the rest of the plant remained under dark conditions. As the actinic light source, either the newly developed LD light described in the previous section or a commercially available LED light (LI-6800-02P, LI-COR, Lincoln, NE, USA) was used under two light colors, 100% red light (“R_100_”) or 80% red and 20% blue light (“R_80_B_20_”).

To investigate the effects of different light sources on photosynthetic induction, time-course responses of photosynthetic parameters were measured. The chamber was maintained at a temperature of 25°C, a RH of 70%, and a PPFD of 950 µmol m^−2^ s^−1^, respectively, and data were collected at 60 s intervals for 60 min under R100 or R80B20 conditions. The measured photosynthetic parameters included CO_2_ assimilation rate (*A*, µmol m^−2^ s^−1^), transpiration rate (*E*, mmol m^−2^ s^−1^), stomatal conductance (*g*_s_, mmol m^−2^ s^−1^). The intrinsic water-use efficiency (iWUE) was calculated using *A* and *g*_s_ as shown in **Equation 1**:

(1) $$iWUE=\;\frac{A}{g_{s}}$$

For the photosynthetic induction curves, the final three data points were taken as representative of the steady-state photosynthetic parameters. Each treatment was replicated at least 4 times using independently grown plants, with one fully expanded leaf measured per plant.

To assess the periodicity of the photosynthetic induction curve, its frequency components were extracted using fast Fourier transform (FFT). The time series of photosynthetic parameters during the induction phase were multiplied by a Hanning window and then subjected directly to FFT in R software (http://www.r-project.org/) using the built−in FFT function. Power spectral density was computed over the frequency band from 0.0003 Hz (the lower limit set by the total observation duration) to 0.006 Hz, and dominant frequencies were identified as peaks.

To obtain the *A-C*_i_ (intercellular CO_2_ concentration) curve and *g*_s_-*C*_i_ curve, the *C*_a_ (ambient CO_2_ concentration) in the gas chamber was sequentially changed to 0, 50, 100, 200, 400, 600, 800, 1000, and 1500 µmol mol^−1^, and *A, g*_s_ and *C*_i_ were measured at each *C*_a_ level. The chamber was maintained at a temperature of 25°C, a RH of 70%, and a PPFD of 300 µmol m^−2^ s^−1^, respectively. Different PPFD levels were used depending on the experimental purpose: high irradiance (950 μmol m^−2^ s^−1^) was applied for photosynthetic induction measurements to detect dynamic stomatal responses, whereas moderate irradiance (300 μmol m^−2^ s^−1^) was used for *A–C*_i_ and *g*_s_–*C*_i_ analyses to ensure stable parameter estimation under non-light-limiting conditions. *V*c_max_, the maximum rate of Rubisco carboxylation, and *J*_max_, the maximum rate of electron transport supporting RuBP (ribulose-1,5-bisphosphate) regeneration, were estimated by fitting the *A-C*_i_ response curves to the Farquhar’s model (Farquhar et al., 1980), using nonlinear least squares (nlsLM) in R software. The Farquhar model of photosynthesis can be formulated as shown in **Equation 2**:

(2) $$A\;=\;min\left(A_{c},\;A_{j}\right)-Rd$$

Where *A*_c_ is Rubisco-limited CO_2_ assimilation rate, *A*_j_ is electron transport-limited CO_2_ assimilation rate, and *R*_d_ is day respiration rate (μmol CO_2_ m^−2^ s^−1^). To ensure stable parameter estimation, *R*_d_ was fixed at 1 following earlier studies for C_3_ plants (e.g., Von Caemmerer, 2000; Yamori et al., 2005). *A* is limited by the smaller of *A*_c_ or *A*_j_, and these two limiting rates can be described in detail by **Equations 3** and **4**:

(3) $$Ac\;=\;\;\frac{\left[V_{cmax}\;\times \;\left(C_{i}\;-\;\Gamma^{*}\right)\right]}{\left[C_{i}\;+\;K_{c}\;\times \;\left(1\;+\;\frac{O}{K_{o}}\right)\right]}$$

(4) $$A_{j}\;=\;\frac{\left[J_{max}\;\times \;\left(C_{i}\;-\;\Gamma^{*}\right)\right]}{\left(4.5\;\times \;C_{i}\;+\;10.5\;\times \;\Gamma^{*}\right)}$$

Where *C*_i_ is intercellular [CO_2_] (μmol mol^−1^), Γ^*^ is CO_2_ compensation point (μmol mol^−1^), K_c_ and K_o_ are Michaelis-Menten constants for CO_2_ and O_2_, and *O* is ambient O_2_ concentration (mmol mol^−1^). Temperature-dependent parameters in the Farquhar model, including Γ^*^, K_c_, and K_o_, were adjusted according to exponential temperature response functions based on activation energies (Bernacchi et al., 2001). *O* was assumed to be 210 mmol mol^−1^. In the *g*_s_–*C*_i_ curve, the intercellular CO_2_ concentration at which stomatal conductance reached its maximum (*g*_s_^*^) was defined as *C*_i_^*^. The regions preceding and following *C*_i_^*^ were designated as the low-*C*_i_ and high-*C*_i_ regions, respectively. Within each region, linear regressions were performed using up to four data points, and the slopes of the regression lines were used as indices of changes in stomatal conductance.

### 2.5 Measurement of stomatal aperture

During the photosynthetic induction experiment described in the previous section, a rice leaf was removed from the gas-exchange cuvette during one of the following time points after the onset of the lighting: 0, 900, 1200, 1800, or 2400 s. Immediately after removal, cyanoacrylate adhesive (Aron Alpha, Toagosei Co., Ltd., Tokyo, Japan) was applied to the abaxial side of the leaf at the irradiated region and left to dry. Once dried, the adhesive film was peeled off to obtain stomatal footprints representing the shape and aperture of stomata. The footprints were observed under a light microscope (CH-40, Olympus Corp., Tokyo, Japan), and at least 200 stomata per sample were visually classified into three categories based on the degree of stomatal opening: closed, partially open, or open (Toda et al., 2018). The relative frequency of each category was then calculated.

### 2.6 Statistical analysis

In this study, statistics were analyzed in R software. Data were analyzed by two-way ANOVA followed by the Tukey’s HSD test (P< 0.05) for comparing between light sources and light color.

## 3 Results

### 3.1 Wavelength characteristics of each light source

**Figure 2:**
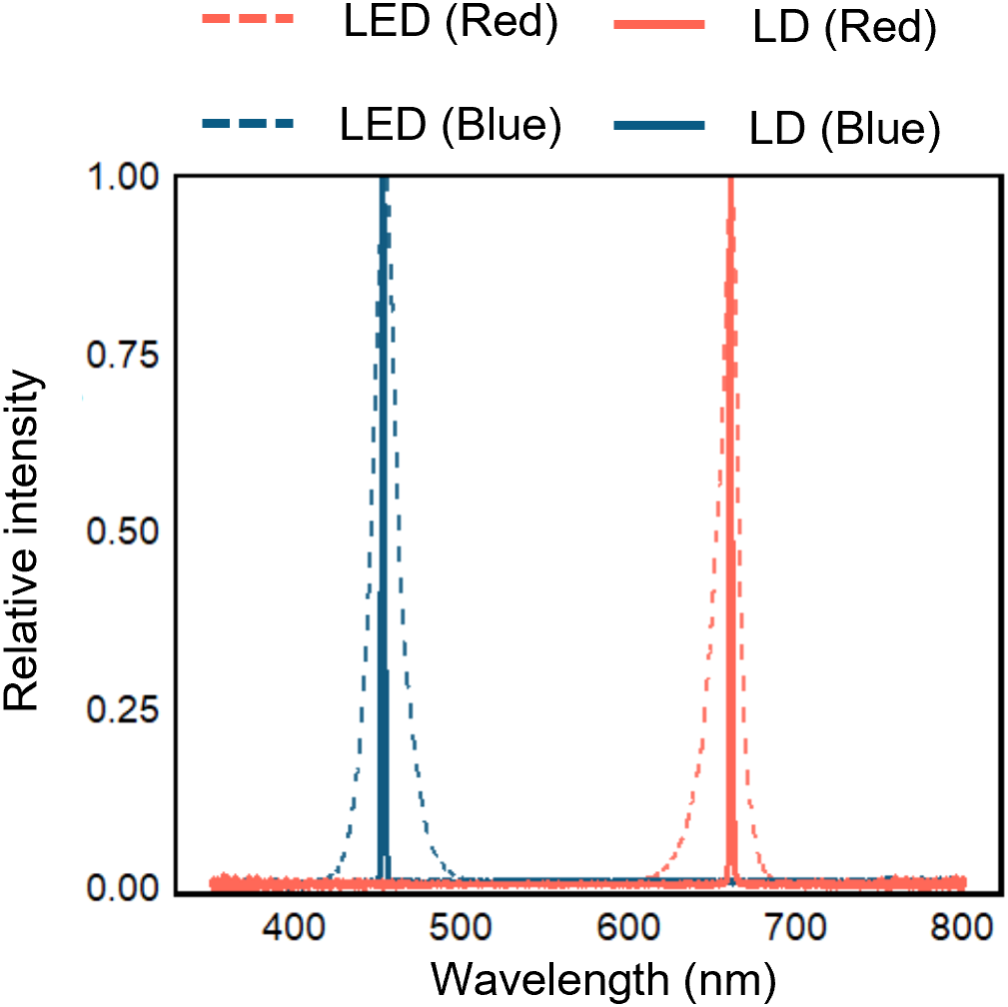
Emitted wavelength by LDs or LEDs. The wavelength spectra for individual irradiation by red and blue LDs and LEDs light are shown. Signal intensity at each wavelength in each spectrum is expressed as a normalized relative value so that the peak intensity is one.

The wavelengths of red and blue LD and LED light sources were measured (**Figure 2**). The emission spectrum of each light source was characterized by its peak wavelength and full width at half maximum (FWHM), which indicates the spectral bandwidth corresponding to half of the maximum intensity. The results showed that the peak wavelength of blue light was 452 nm for the LD and 454 nm for the LED. Similarly, the red LD and LED exhibited peak wavelengths of 660 nm and 661 nm, respectively. Thus, for both blue and red light, the peak wavelengths were nearly identical regardless of the light source. When the spectral width was defined as FWHM, the blue LD had a FWHM of 2.17 nm and the red LD 1.80 nm, whereas the blue and red LEDs exhibited much broader FWHM of 17.1 nm and 13.6 nm, respectively (**Table 1**).

**Table 1:** Full width at half maximum for each light source.

Light color	FWHM^1^	
	LD (nm)	LED (nm)
Blue	2.17	17.1
Red	1.80	13.6

FWHM^1^ stands for ‘full width at half maximum’ and refers to the width between the two wavelengths on either side of the peak at which the intensity equals half of the maximum.

### 3.2 Steady-state photosynthetic parameters under LDs and LEDs

**Figure 3:**
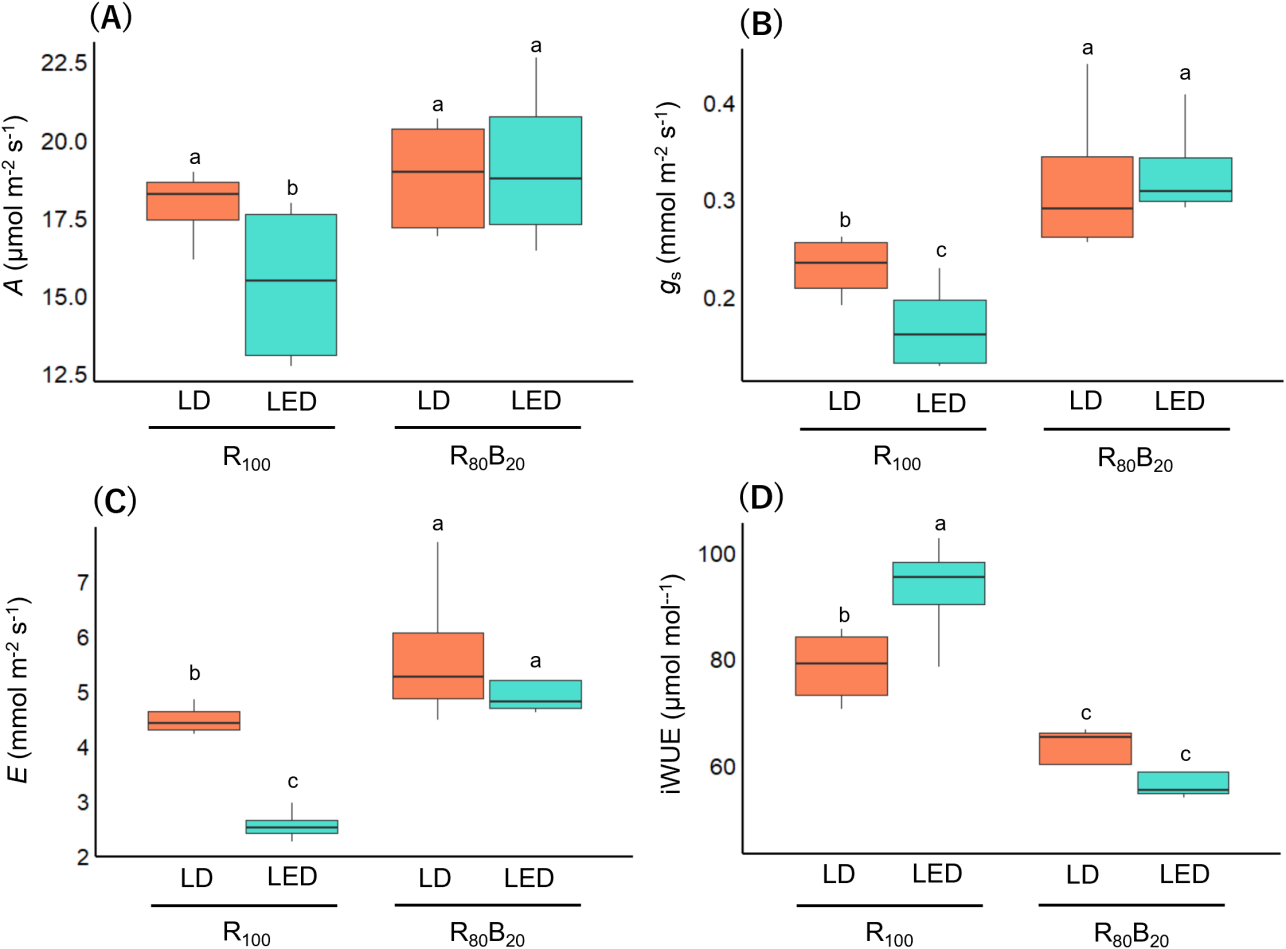
Comparison of steady−state photosynthetic parameters under different light sources and light color. For each photosynthetic parameter, the final three measurements of its time−response curve were defined as steady−state values, yielding 12 data points per each treatment across four experimental replicates (n = 12). Each panel shows **(A)** CO_2_ assimilation rate, **(B)** stomatal conductance, **(C)** transpiration rate and **(D)** intrinsic water use efficiency at 400 µmol mol^–1^ CO_2_. R_100_ denotes irradiation with red light only, whereas “R_80_B_20_” denotes simultaneous irradiation with red and blue light at an 80:20 ratio. For each photosynthetic parameter, multiple comparisons among treatments following two-way ANOVA were conducted using the Tukey HSD, and statistically significant differences were indicated (*P* < 0.05). Light color significantly affected all photosynthetic parameters (*P* < 0.01), while significant interactions between light source and light color were observed for all parameters (*A*: *P* = 0.012; *g*_s_: *P* = 0.017; *E*: *P* < 0.01; iWUE: *P* < 0.001). The main effect of light source was significant only for transpiration rate (*P* < 0.001) and intrinsic water use efficiency (*P* < 0.01).

In rice under R_100_ LD, steady‐state net CO_2_ assimilation rate (*A*) was significantly higher than under R_100_ LED (**Figure 3A**), and stomatal conductance (*g*_s_) and transpiration (*E*) rate were elevated (**Figures 3B, C**). Because the relative increase in stomatal conductance exceeded that of net CO_2_ assimilation rate, intrinsic water‐use efficiency (iWUE) was reduced under R_100_ LD (**Figure 3D**). By contrast, when rice was illuminated with R_80_B_20_ LD versus LED, there were no significant differences in *A*, *g*_s_, *E*, or iWUE (**Figures 3A–D**). Comparison of absolute values across all treatments revealed that *A* under R_80_B_20_ LD and LED, and R_100_ LD did not differ and were uniformly higher than under R_100_ LED, which exhibited the lowest *A* (**Figure 3A**). Likewise, *g*_s_ and *E* were highest and equivalent under R_80_B_20_ LD and LED, intermediate under R_100_ LD, and lowest under R_100_ LED (**Figures 3B, C**).

### 3.3 Evaluation of periodicity in photosynthetic induction curves

**Figure 4:**
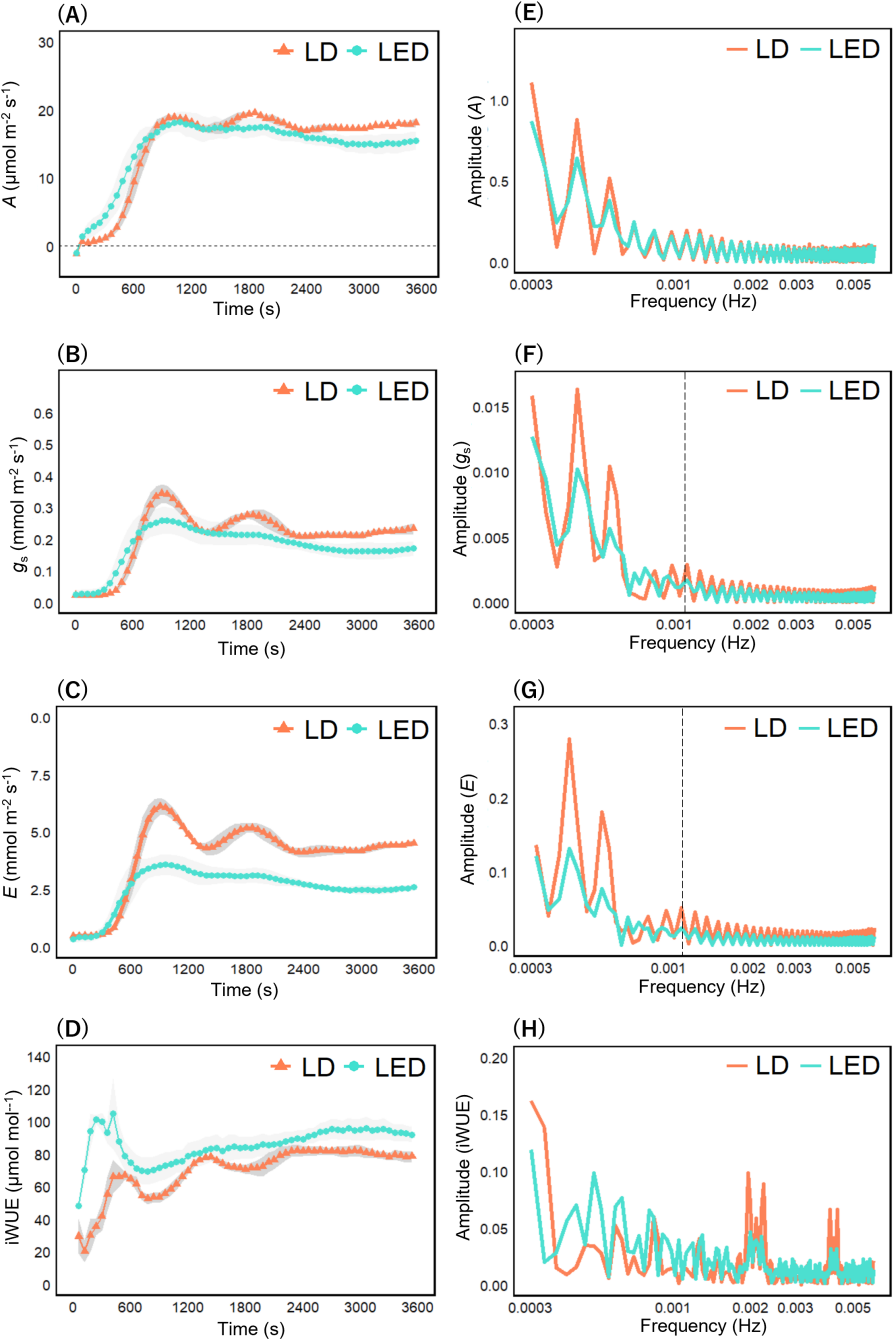
Photosynthetic induction curves and their frequency spectrums under R_100_ illumination. Induction curves of photosynthetic parameters in rice under R_100_ illumination **(A–D)**, and their corresponding frequency spectrums **(E–H)** by using a fast Fourier transform (FFT). Each panel shows induction curves of **(A)** CO_2_ assimilation rate, **(B)** stomatal conductance, **(C)** transpiration rate, and **(D)** intrinsic water use efficiency, frequency spectrums of **(E)** CO_2_ assimilation rate, **(F)** stomatal conductance, **(G)** transpiration rate and **(H)** intrinsic water use efficiency. The first two points in **(D)** were excluded because they had negative values. The photosynthetic parameters were recorded every 60 s at an irradiance of 950 μmol photons m^–2^ s^–1^ until 3600 s and expressed as the mean ± standard error (n = 4). Dashed lines in **(F, G)** marked the frequency components specific to LDs relative to LEDs.

Next, we compared the induction curves of each photosynthetic parameter. In rice under R_100_ LD, periodic oscillations over time were observed most prominently in *g*_s_ and *E* compared with R_100_ LED (**Figures 4B, C**). *A* and iWUE, on the other hand, exhibited weaker or less distinct oscillatory tendencies compared with the stomatal parameters (**Figures 4A, B**). To quantify these periodicities, we applied fast Fourier transform (FFT) to the induction curves and found that *A* and iWUE showed only minor differences in their frequency spectra between R_100_ LD and R_100_ LED (**Figures 4E, H**), whereas *g*_s_ and *E* under R_100_ LD exhibited a distinct frequency component at 1.12 × 10^−3^ Hz (≈ 900 s) that was not observed under R_100_ LED (**Figures 4F, G**).

**Figure 5:**
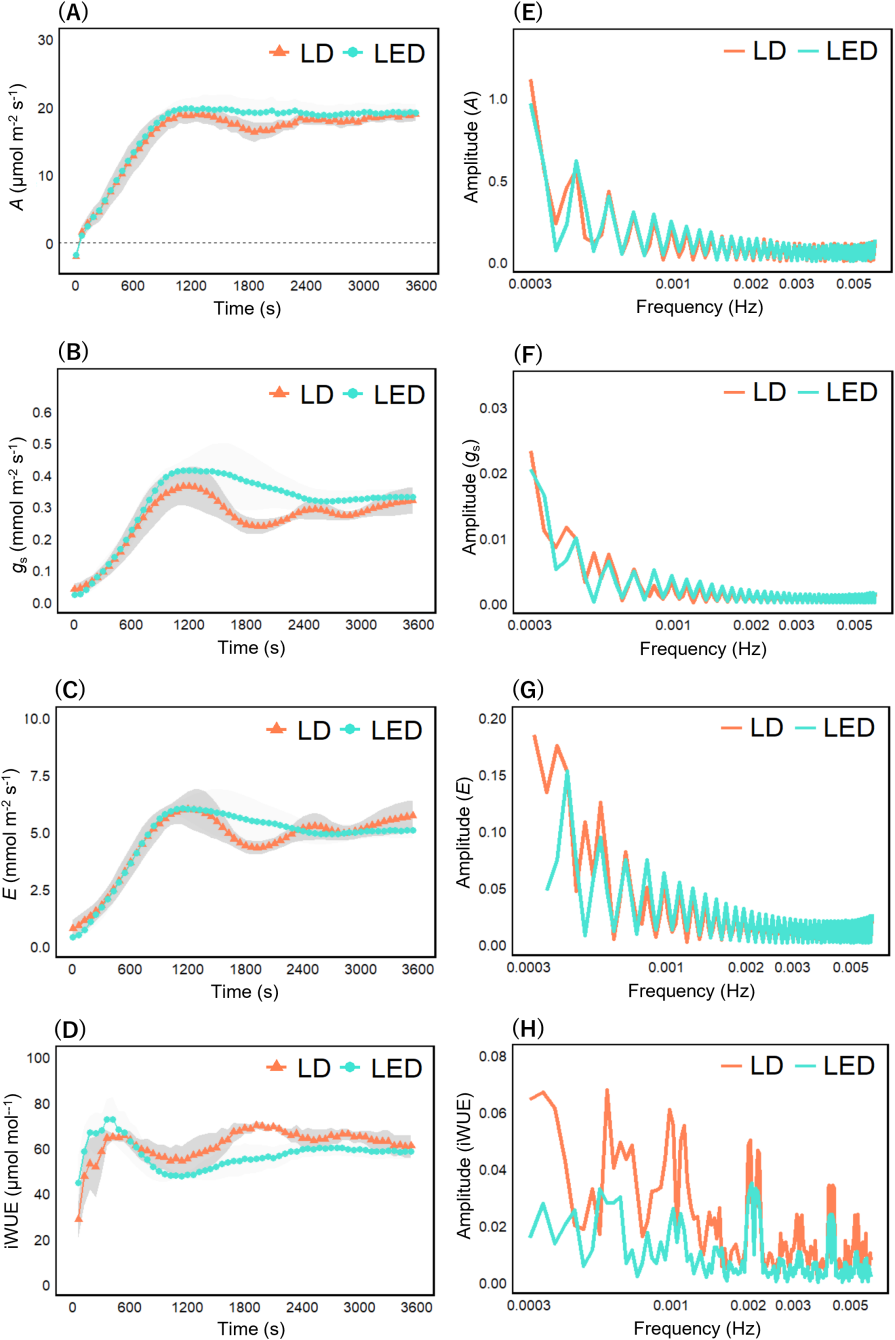
Photosynthetic induction curves and their frequency spectrums under R_80_B_20_ illumination. Induction curves of photosynthetic parameters in rice under R_100_ illumination **(A–D)**, and their corresponding frequency spectrums **(E–H)** by using a fast Fourier transform (FFT). Each panel shows induction curves of **(A)** CO_2_ assimilation rate, **(B)** stomatal conductance, **(C)** transpiration rate, and **(D)** intrinsic water use efficiency, frequency spectrums of **(E)** CO_2_ assimilation rate, **(F)** stomatal conductance, **(G)** transpiration rate and **(H)** intrinsic water use efficiency. The first two points in **(D)** were excluded because they had negative values. The photosynthetic parameters were recorded every 60 s at an irradiance of 950 μmol photons m^–2^ s^–1^ until 3600 s and expressed as the mean ± standard error (n = 4).

Under R_80_B_20_ illumination, as in the R_100_ condition, periodic oscillations in the induction curves of *g*_s_ and *E* were observed under LDs but not under LEDs (**Figures 5B, C**). However, these oscillations appeared to have longer periods than those under R_100_ LDs (**Figures 5B, C**). In contrast, oscillatory behavior in *A* and iWUE was much less apparent than in *g*_s_ and *E* (**Figures 5A, D**). To compare these responses with those under LEDs, we again performed FFT analysis. The FFT results showed no substantial differences in oscillation amplitude of *A*, *g*_s_, or *E* between R_80_B_20_ LD and LED treatments (**Figures 5E–G**), whereas the amplitude of iWUE was greater under R_80_B_20_ LDs (**Figure 5H**). Moreover, the frequency component around 900 s that was evident in *g*_s_ and *E* under R_100_ LDs was absent under R_80_B_20_ LD (**Figures 5F, G**).

### 3.4 Visualization of stomatal aperture

**Figure 6:**
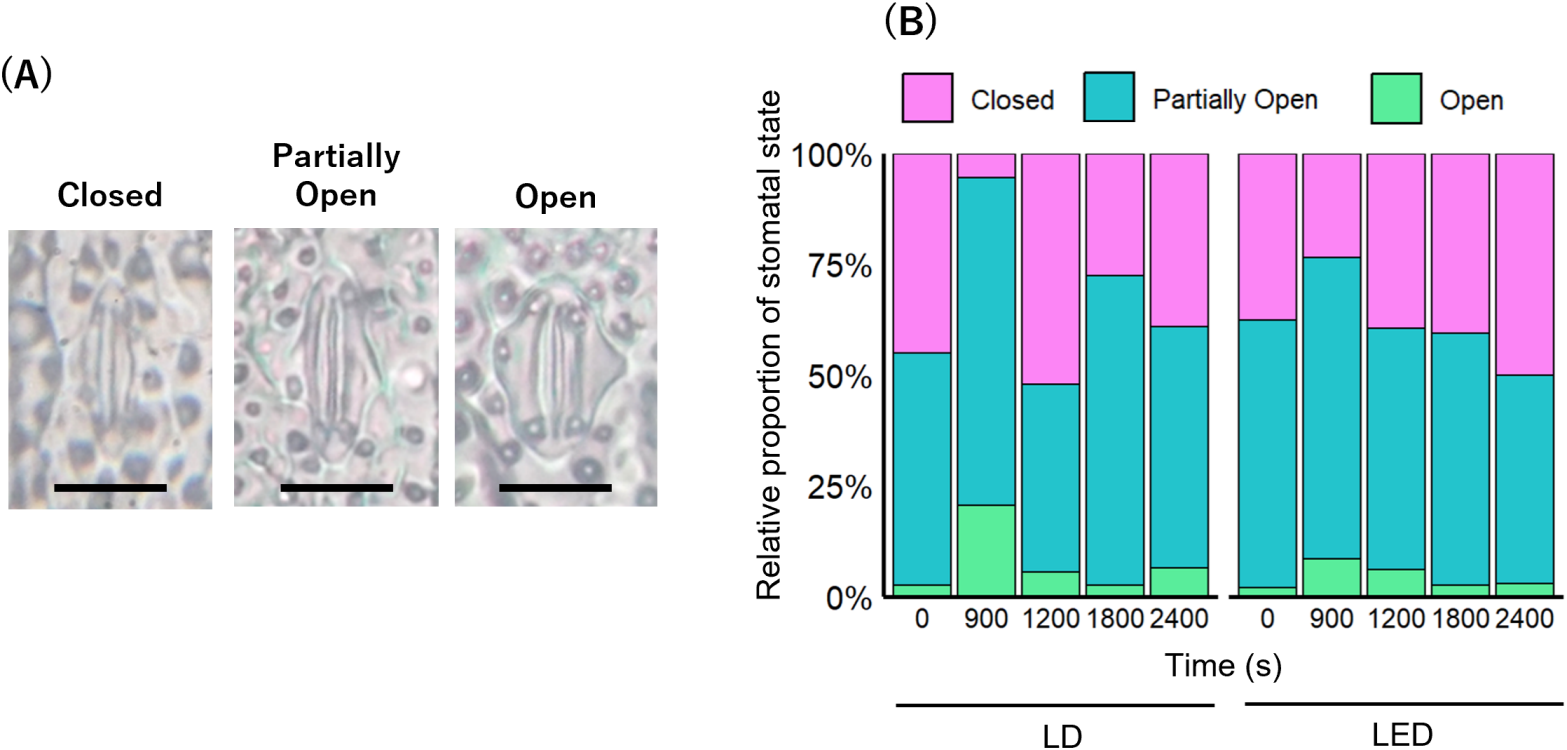
The stomatal aperture under LD and LED illumination. **(A)** Three optical microscope images showing the stomatal states (Closed, Partially Open, Open). Bar = 20 µm. **(B)** Relative proportion of stomatal states over time for each light source (n ≧ 200).

Because *g*_s_ and *E* showed dynamic temporal fluctuations under R_100_ LD (**Figures 4B, C**), we used microscopy to visually examine stomatal morphology. Following a classification of Toda et al. (2018), stomata were divided into three categories, “Closed”, “Partially Open”, and “Open” (**Figure 6A**). We then tracked the relative proportion of each category over time in rice under R_100_ LDs versus LEDs. Under R_100_ LDs, the percentage of open stomata (“Partially Open” + “Open”) increased to 94.5% at 900 s and to 72.5% at 1800 s (**Figure 6B**). By contrast, under R_100_ LEDs the proportion of open stomata peaked at 76.5% at 900 s and then gradually declined to 50.0% by 3600 s (**Figure 6B**). These shifts in open‐stomata ratio reflected the time courses of *g*_s_ and *E* observed under R_100_ LDs and LEDs (**Figures 4B, C**).

### 3.5 CO_2_ response and photosynthetic capacity parameters under LD and LED

**Figure 7:**
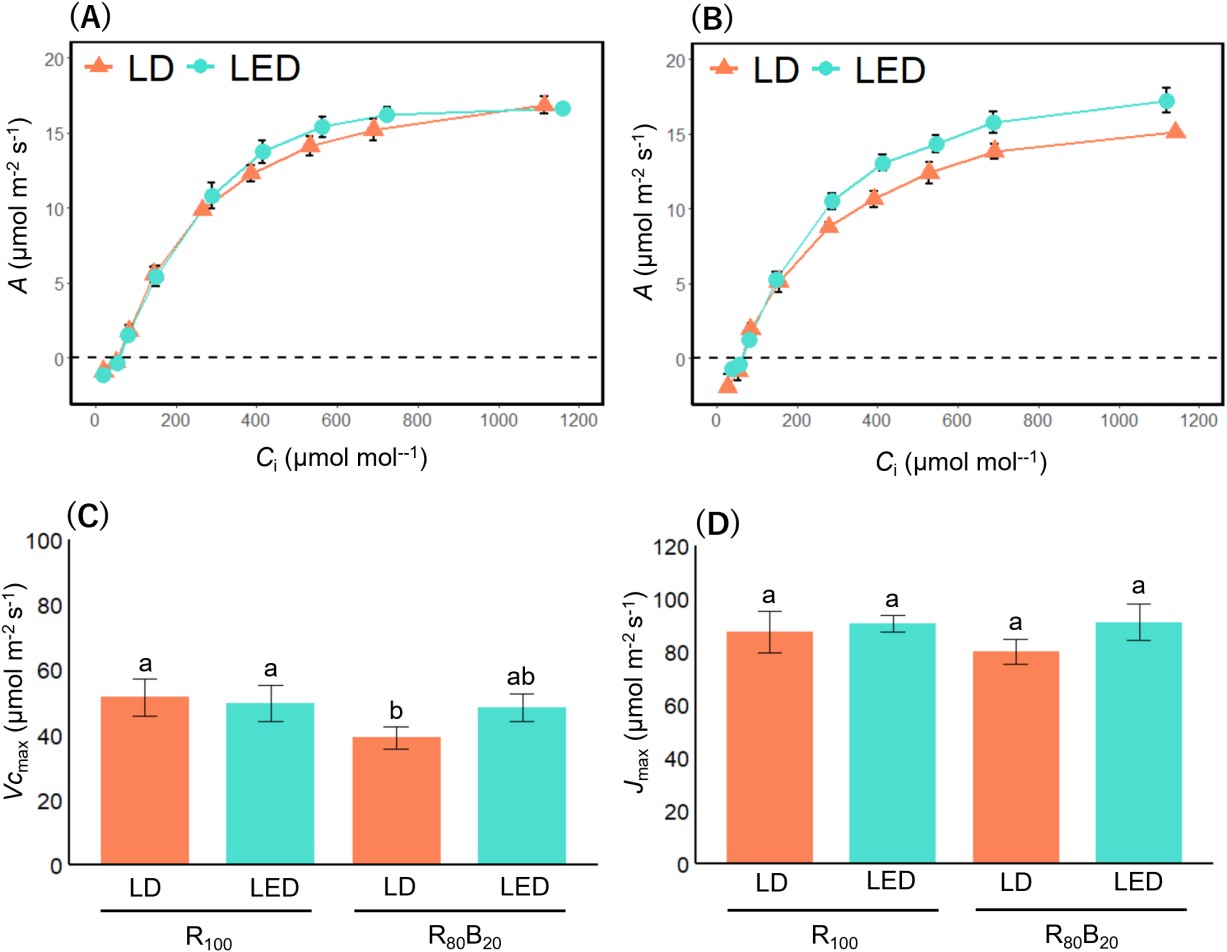
*A*–*C*_i_ curves and photosynthetic capacity parameters. The *A*–*C*_i_ curves and the Farquhar-model-derived photosynthetic parameters under LED and LD illumination are shown. Each panel shows **(A)** *A*–*C*_i_ curve under R_100_, and **(B)** under R_80_B_20_, **(C)** maximum carboxylation rate (*V*cmax) and **(D)** maximum electron transport rate (*J*max). The *A*–*C*_i_ curves were measured at an irradiance of photons m^–2^ s^–1^ until 3600 s. R_100_ denotes irradiation with red light only, whereas R_80_B_20_ denotes simultaneous irradiation with red and blue light at an 80:20 ratio. Values are expressed as the mean ± standard error (n ≧ 4). For each photosynthetic capacity parameter, multiple comparisons among treatments following two-way ANOVA were conducted using the Tukey HSD, and statistically significant differences were indicated (*P* < 0.05). Light color and its interaction with light source significantly affected *V*cmax (light color: *P* = 0.016; light color × light source: *P* = 0.03), whereas only light source had a significant effect on *J*max (*P* = 0.02).

By varying ambient CO_2_ (*C*_a_) and measuring *A*, *g*_s_ and intercellular CO_2_ concentration (*C*_i_), we generated both *A*-*C*_i_ and *g*_s_-*C*_i_ curves to probe stomatal dynamics under LD illumination. Under R_100_ LDs versus LEDs, *A*-*C*_i_ responses did not differ significantly (**Figure 7A**), whereas under R_80_B_20_ LDs, *A* remained relatively lower as *C*_i_ increased compared with R_80_B_20_ LEDs (**Figure 7B**). To discover why CO_2_ assimilation was depressed under R_80_B_20_ LDs, we estimated photosynthetic capacity parameters, maximum carboxylation rate (*V*cmax) and maximum electron transport rate (*J*max) from the *A*-*C*_i_ curves by using Farquhar’s model (Farquhar et al., 1980). *V*cmax values tended to cluster into higher (R_100_ LDs and LEDs) and lower (R_80_B_20_ LDs) groups, with statistical overlap leaving R_80_B_20_ LEDs intermediate (**Figure 7C**). In contrast, *J*max in rice grown under R_80_B_20_ LD was comparable to that under the other light treatments (**Figure 7D**).

**Figure 8:**
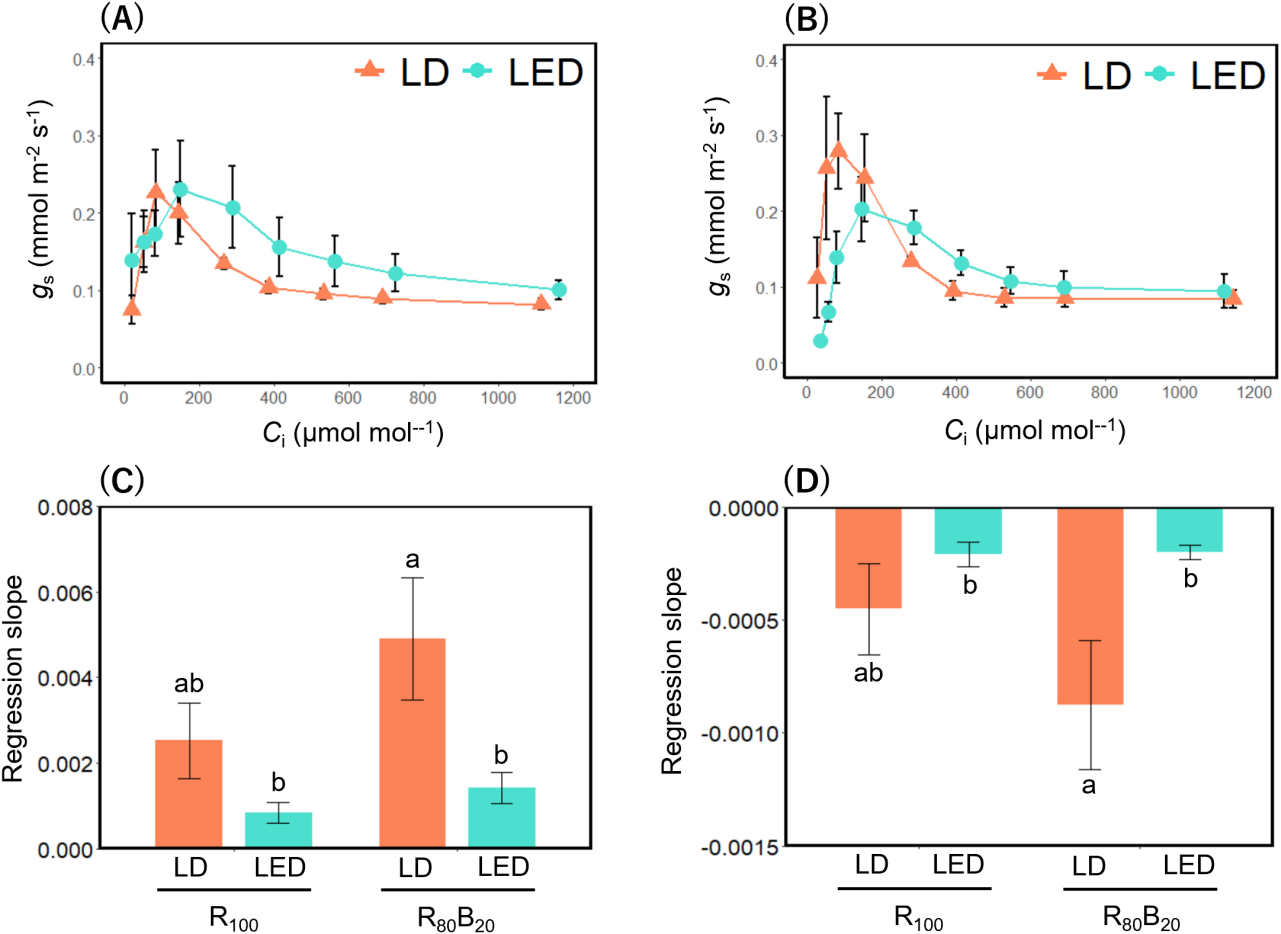
*g*_s_–*C*_i_ curves and their linear regression slopes. The *g*_s_–*C*_i_ curves and the linear regression slopes before and after the *C*_i_^*^ peak under LED and LD illumination are shown. The *C*_i_ at which *g*_s_ reaches its maximum is denoted as *C*_i_^*^. Each panel shows **(A)** *g*_s_–*C*_i_ curve under R_100_, and **(B)** under R_80_B_20_, **(C)** regression slope before *C*_i_^*^, and **(D)** regression slope after *C*_i_^*^. R_100_ denotes irradiation with red light only, whereas R_80_B_20_ denotes simultaneous irradiation with red and blue light at an 80:20 ratio. Values are expressed as the mean ± standard error (n ≧ 4). For each regression slope, multiple comparisons among treatments following two-way ANOVA were conducted using the Tukey HSD, and statistically significant differences were indicated (*P* < 0.05). The regression slopes both before and after *C*i^*^ were significantly affected by light source (before *C*i^*^: *P* < 0.01; after *C*i^*^: *P* = 0.012), whereas neither light color nor its interaction with light source had significant effects on either regression slope (*P* ≧ 0.098).

By varying *C*_a_, we obtained *g*_s_ corresponding to *C*_i_. Under the R_100_ condition, the maximum *g*_s_ (*g*_s_*) did not differ between LD and LED treatments, but the *C*_i_ corresponding to *g*_s_* (*C*_i_*) was lower under LDs than LEDs (**Figure 8A**). Under the R_80_B_20_ condition, *C*_i_* was also lower under LDs, and *g*_s_* was greater under LDs than LEDs (**Figure 8B**). To quantify stomatal responsiveness to CO_2_, linear regressions were performed before and after *C*_i_*, and the regression slopes were extracted. In both the before *C*_i_* and after *C*_i_* regions, LD treatments exhibited significantly steeper slopes than LEDs treatments, with a particularly pronounced difference under the R_80_B_20_ condition (**Figures 8C, D**).

## 4 Discussion

### 4.1 Contribution of LDs to photosynthesis via red light-dependent stomatal response

In the present study, *g*_s_ and *E* of rice grown under R_100_ LDs were higher than those under R_100_ LEDs, but lower than those under R_80_B_20_ in both LD and LED conditions (**Figures 3B, C**). By contrast, *A* under R_100_ LDs remained high and did not differ significantly from that under R_80_B_20_ (**Figure 3A**). These findings suggest that stomatal opening in rice under R_100_ LDs may be driven predominantly by a red light–dependent mechanism.

Extensive LED-based studies have shown that supplementing red light with even a small proportion of blue light markedly enhances photosynthetic parameters such as *A* and *g*_s_ compared with monochromatic red light (Hogewoning et al., 2010; Savvides et al., 2012; Wang et al., 2016). For instance, Savvides et al. (2012) demonstrated that providing cucumber leaves with a mixture of red LED and 30% blue LED increased stomatal conductance to more than three times that under red light alone, accompanied by a rise in photosynthetic rate. Similarly, Hogewoning et al. (2010) reported that adding as little as 7% blue light to red illumination more than doubled the maximal photosynthetic rate relative to pure red light. These studies underpin the prevailing view that stomatal opening is primarily driven by blue light.

However, in our study, *g*_s_ under R_100_ LDs exceeded that under R_100_ LEDs (**Figure 3B**), and *A* under R_100_ LDs was comparable to that under R_80_B_20_ (**Figure 3A**). These observations indicate that stomatal opening in rice under R_100_ LDs is likely mediated mainly by red light. Red light–dependent photosynthesis in mesophyll or guard cells has been shown to trigger stomatal responses independently of blue-light signaling (Baroli et al., 2008; Ando and Kinoshita, 2018; Bernardo et al., 2023). Such mechanisms may have been activated more strongly under LD than LED illumination. The possibility that red LDs promotes stomatal opening has also been proposed previously (Li et al., 2025). Nevertheless, LDs differ from LEDs in several optical properties—monochromaticity, coherence, and beam collimation among them—and the physiological effects of such extremely narrowband illumination remain poorly understood and have not been systematically investigated, making it unclear which of these characteristics contribute to enhanced stomatal opening.

### 4.2 LD illumination stimulates biological oscillations

In this study, rice under R_100_ LDs exhibited pronounced periodicity in *g*_s_ and *E* (**Figures 4B, C, F, G**), although this periodicity was diminished in rice under R_80_B_20_ (**Figures 5B, C, F, G**). In contrast, under LED illumination, *g*_s_ and *E* increased once after the onset of measurement but subsequently declined, irrespective of whether blue light was present (**Figures 4B, C**; **Figure 5B, C**). Furthermore, the proportion of “Partially Open” and “Open” stomata in rice under R_100_ LD and LED conditions (**Figure 6B**) closely mirrored the rise-and-fall patterns observed in the induction curves of *g*_s_ and *E* (**Figures 4B, C**; **5B, C**). Collectively, these observations indicate that red LDs promote rapid and periodic stomatal opening and closing in rice, whereas blue LDs appear to suppress stomatal dynamics.

Many physiological processes display oscillations on time scales ranging from 10 min to 24 h or longer, referred to as “biological rhythms” (McClung, 2006; Damineli et al., 2022). Stomatal movements are also strongly influenced by such rhythms: oscillations in stomatal conductance and transpiration rate with periods of tens of minutes, classically described as oscillatory stomatal behavior (Farquhar and Cowan, 1974) or transpiration oscillation (Johnsson et al., 1979), are sometimes referred to as “ultradian rhythms” in more recent studies (Zait et al., 2017; Peak et al., 2023). Our study indicated that stomatal oscillations during photosynthetic induction in rice were most pronounced under red LDs, whereas these oscillations were suppressed under blue LDs. Periodic Ca²^+^ oscillations in guard cells are considered a primary driver of stomatal ultradian rhythms, as they rhythmically regulate ion transporters such as H^+^-ATPases and K^+^ channels (Yang et al., 2005; Damineli et al., 2022), giving rise to membrane-potential oscillations. These electrical oscillations interact with hydraulic cycles produced by transpiration-driven declines and subsequent recovery of leaf water potential (Steppe et al., 2006), together generating the characteristic periodic opening and closing of stomata. We confirmed that applying Ca²^+^ to the leaf surface of lettuce enhanced stomatal conductance and transpiration rate under red LD illumination compared with plants without Ca²^+^ application (data not shown). Collectively, these results suggest that red LDs may modulate the amplitude and persistence of stomatal oscillations during photosynthetic induction through changes in intracellular Ca²^+^ concentrations in leaves. Although a LED-based study has suggested that red light–dependent stomatal opening requires the activation of ion transporters such as H^+^-ATPase and K^+^ channels (Ando and Kinoshita, 2018), it remains unclear why red LDs is able to activate these mechanisms more strongly.

### 4.3 Blue LDs present both advantages and disadvantages for photosynthesis

In rice under R_80_B_20_ LDs, stomatal movements were far more pronounced at lower *C*_i_, and the amplitude of those movements exceeded that of the other three treatment groups (**Figures 8A, B**). In other words, despite low *C*_i_, plants under R_80_B_20_ LDs opened their stomata more widely and took up CO_2_ more effectively. This behavior follows the basic blue‐light-dependent stomatal opening mechanism: blue light absorbed by the phototropin receptors phot1 and phot2 in guard cells activates plasma membrane H^+^-ATPase and K^+^ channels, driving stomatal aperture (Inoue and Kinoshita, 2017). Moreover, because *g*_s_^*^ under R_80_B_20_ LDs exceeded that under R_100_ LDs (**Figure 3B**), the blue LDs appear to act additively with red LDs to enhance stomatal opening.

A theoretical model predicts that when stomatal conductance responds strongly even to small changes in *C*_i_, CO_2_ assimilation capacity should increase accordingly (Medlyn et al., 2011). However, contrary to this model, rice under R_80_B_20_ LDs exhibited lower CO_2_ assimilation rates across rising *C*_i_ than the other three treatments (**Figures 7A, B**). In addition, *V*cmax under R_80_B_20_ LDs tended to be lower than that of the other treatments, whereas *J*max remained unchanged (**Figures 7C, D**). These results imply that blue LDs may impose a non-stomatal limitation on photosynthesis, despite promoting blue‐light‐dependent stomatal opening. This negative impact may arise from the interaction of LD’s inherent coherence and the heterogeneous absorption of blue light in the leaf. When coherent LD light illuminates a leaf, speckle contrast creates a patchwork of high‐ and low‐intensity zones determined by surface microtopography (Zhong et al., 2013). Thus, transmitted LD photons may interfere to form localized light‐intensity “hot” and “cold” spots within the tissue. Furthermore, blue light is absorbed almost entirely (≈98%) in the palisade mesophyll, whereas red light penetrates both palisade and spongy mesophyll layers (Vogelmann and Evans, 2002). Concentrated absorption of blue light in the palisade has been shown to slow electron transport and reduce photosynthetic rate by about 11% (Earles et al., 2017). It should be emphasized that this proposed mechanism remains speculative and requires direct experimental validation, for example through imaging of intra-leaf light absorption and distribution under LD versus LED illumination. Taken together, under blue LDs, its coherent speckle‐induced heterogeneity and localized overabsorption in the palisade are likely to suppress overall photosynthetic efficiency.

### 4.4 Red LDs as cultivation lighting and industrial potential of LDs

In this study, we evaluated photosynthetic parameters of rice under blue LDs with a peak wavelength of 452 nm and red LDs with a peak wavelength of 660 nm, both of which exhibit much narrower spectral bandwidths than LEDs (**Figure 2**; **Table 1**). In contrast, lighting systems currently used in PFALs predominantly rely on white or purple LEDs, which provide mixed spectra composed of multiple monochromatic components rather than narrowband light sources (Rihan et al., 2022; Yano et al., 2023). Therefore, when considering LDs as alternative light sources for PFALs, it is necessary to systematically evaluate which wavelengths and spectral characteristics confer physiological advantages, including scenarios in which LDs of different colors are combined.

Under both LD and LED conditions, rice under R_100_ lighting exhibited significantly higher iWUE than rice under R_80_B_20_ lighting (**Figure 3D**). Blue light is known to accelerate stomatal and photosynthetic induction; however, under steady-state conditions, increases in stomatal conductance can exceed gains in CO_2_ assimilation, potentially resulting in reduced water-use efficiency (Matthews et al., 2020). Such mechanisms likely underlie the lower iWUE observed in rice under R_80_B_20_ conditions compared with R_100_ LDs or LEDs. In addition, rice under R_100_ LD, but not under R_100_ LED, maintained CO_2_ assimilation rates comparable to those observed under R_80_B_20_ LDs or LEDs (**Figure 3A**). These results indicate that rice leaves illuminated with red LDs peaking at 660 nm can achieve high photosynthetic rates while minimizing water loss, suggesting that 660 nm red LDs may represent an effective cultivation light source for PFALs.

Nevertheless, because the red LDs used in this study had an extremely narrow spectral width (**Table 1**), it remains uncertain whether similar physiological responses would be observed under red LDs with different peak wavelengths or broader spectral widths. Consistent with this concern, tobacco grown under red LDs peaking at 660, 664, and 673 nm exhibited maximal net photosynthetic rates at 660 nm, with a sharp decline at longer wavelengths (Li et al., 2025). Moreover, *A. thaliana* cultivated under a combination of a 671 nm red LDs and a 473 nm blue LDs showed reduced chlorophyll content and dry mass compared with plants grown under white LED illumination (Ooi et al., 2016). Taken together, these findings indicate that while red LDs peaking at 660 nm can simultaneously enhance water-use efficiency and photosynthetic performance, their effectiveness is strongly dependent on spectral properties, underscoring the need for precise spectral control when applying LDs to PFAL systems.

Beyond spectral optimization, practical implementation of LD lighting in cultivation systems also requires consideration of engineering constraints such as energy efficiency and water availability. It should be noted that energy consumption and total operational costs of LD- and LED-based lighting systems were not directly evaluated in this study and therefore remain topics for future investigation. Future studies should therefore integrate direct measurements of electrical power input with photosynthetic performance to assess energy-use efficiency under practical PFALs. Advances in manufacturing processes and yield improvements associated with large-scale production have substantially reduced the cost of LED lighting (Tsao et al., 2010), suggesting that LEDs generally require lower initial investment than LDs as cultivation light sources. In contrast, LDs can deliver narrowband light closely matching chlorophyll absorption peaks, a property that has been suggested to improve the efficiency with which light energy is utilized for photosynthesis. For example, red LD illumination at 660 nm has been shown to significantly enhance photosynthetic capacity compared with red LEDs of the same peak wavelength (Li et al., 2025).

High water-use efficiency under LD illumination may be particularly advantageous in cultivation environments where water availability is severely constrained, such as space. On the International Space Station (ISS), more than 90% of consumed water is recycled (Ichimura and Yamashiki, 2025), whereas the remaining fraction must be resupplied from Earth via cargo missions every one to two months (Lynch et al., 2024). In such environments, cultivation systems that maximize water-use efficiency are essential. As demonstrated in this study, rice under R_100_ LDs exhibited higher water-use efficiency while maintaining photosynthetic rates comparable to those under LED illumination (**Figures 3A, D**), suggesting that LD lighting may serve as a promising light source candidate for extreme cultivation environments, including space agriculture, which lies along the conceptual extension of PFALs.

## 5 Conclusion

In this study, to explore the feasibility of LDs as cultivation light sources in PFALs, we built an LD lighting unit that can be mounted on a commercial gas-exchange system and delivers simultaneous red and blue illumination. Using this unit, we obtained photosynthetic induction curves, *A*-*C*i curves, and *g*_s_-*C*i curves to evaluate the effects of LD lighting on rice photosynthesis. Under red LDs, rice maintained high WUE while exhibiting higher stomatal conductance, transpiration rate, and CO_2_ assimilation rate than under red LED light. We also found that stomatal conductance and transpiration rate showed temporal oscillations with a period of ≈900 s, matching the oscillatory pattern of stomatal aperture. In contrast, under mixed red/blue LDs, stomatal conductance, transpiration, and CO_2_ assimilation did not differ from those under LEDs. Moreover, photosynthetic capacity parameters tended to be lower than under red LDs or LEDs, whereas stomatal responsiveness to changes in CO_2_ concentration was greater than with red LDs or LEDs alone. Taken together, these results indicate that red LDs are a promising PFALs cultivation light source capable of balancing WUE and productivity, while the use of blue LDs remains open to debate. While the beneficial effects of red LDs on plant growth have been reported in previous studies, the present study provides new insight into the underlying physiological mechanisms, particularly in terms of stomatal dynamics and photosynthetic induction responses. Specifically, monochromatic red LDs show promise for enhancing photosynthetic rate and water-use efficiency, whereas blue LDs introduce a more complex trade-off between enhanced stomatal responsiveness and potential non-stomatal limitations to photosynthesis. Building on the potential advantages of red LDs in plant physiology, our future work will focus on developing an LD-based cultivation system that reduces irradiation costs while maintaining high productivity.

## Data Availability

The raw data supporting the conclusions of this article will be made available by the authors, without undue reservation.
